# Profil épidémiologique, clinique et échographique des patients opérés pour un rétrécissement aortique sous-valvulaire dans la région de Sfax (Tunisie) et facteurs associés à la récidive post-opératoire: étude observationnelle

**DOI:** 10.11604/pamj.2022.41.288.28561

**Published:** 2022-04-08

**Authors:** Amine Bahloul, Selma Charfeddine, Dorra Abid, Rania Hammami, Leila Abid, Samir Kammoun

**Affiliations:** 1 Service de Cardiologie, Centre Hospitalo-Universitaire Hèdi Chaker, Sfax, Tunisie

**Keywords:** Rétrécissement aortique sous-valvulaire, obstacle sous aortique, cardiopathie congénitale, diaphragme sous-aortique, Subvalvular aortic stenosis, subaortic obstacle, congenital heart disease, aortic diaphragm

## Abstract

Le rétrécissement aortique sous-valvulaire reste difficile à prendre en charge, du fait du caractère évolutif imprévisible de la sténose et du taux de récidive élevé après le traitement chirurgical. Les objectifs de notre travail étaient de décrire le profil des patients opérés pour un rétrécissement aortique sous valvulaire et d´analyser les facteurs associés à la récidive post-opératoire de l´obstacle sous aortique. Étude observationnelle portant sur tous les cas de rétrécissement aortique sous-valvulaire opérés et colligés dans le service de cardiologie de l'hôpital universitaire de Sfax entre janvier 2010 et décembre 2020. Notre série comporte 28 patients avec une prédominance masculine (64,29%, n=18). Au moment du diagnostic, l´âge moyen était 6,82 (±4,84) ans et les symptômes étaient présents chez 19 patients (67,85%). A l´échocardiographie, un gradient maximal sous-aortique ≥ 50 mmHg était présent chez 23 patients (82,14 %). Les malformations cardiovasculaires associées à la sténose sous valvulaire étaient observées chez 16 patients (57,14%). L´âge moyen au moment de la chirurgie était 10,43 (±7,08) ans. La résection de la membrane sous-aortique était la technique la plus fréquemment utilisée (46,4%, n=13). Elle a été associée à une myomectomie septale chez 8 patients (28,6%). La mortalité opératoire était nulle. En post-opératoire, la présence d´un gradient résiduel ≥ 30 mmHg était notée chez 8 patients (28,6%). La récidive était observée chez 7 patients (25%) parmi lesquels 6 ont bénéficié d´une réintervention. Dans l´analyse multivariée, seule la présence d´un gradient résiduel post opératoire était significativement associée à la récidive (p=0,030, OR=33,785, IC 95%: 1,398-816,754). Malgré l´âge tardif des patients au moment du diagnostic et de la chirurgie, nos résultats étaient favorables à court terme mais la récidive était fréquente à long terme. Ceci souligne l´intérét d´assurer un suivi régulier, clinique et échographique pré et postopératoire de ces patients.

## Abstract (en)

Subvalvular aortic stenosis is difficult to manage due to the evolutionary unpredictability of stenosis and a high recurrence rate after surgical treatment. The purpose of this study is to describe the profile of patients undergoing surgery for the treatment of aortic subvalvular stenosis and to investigate factors associated with post-operative recurrence of the subaortic obstacle. We conducted an observational study of all patients operated for subvalvular aortic stenosis, whose data were collected in the Department of Cardiology of the Sfax University Hospital between January 2010 and December 2020. The study involved 28 patients, with predominance of male sex (64.29%, n=18). At diagnosis, the mean age was 6.82 (±4.84) years and 19 patients (67.85%) had symptoms. On echocardiography, maximal subaortic gradient ≥50 mmHg was found in 23 patients (82.14%). Cardiovascular malformations associated with subvalvular stenosis were found in 16 patients (57.14%). The average age of patients at the time of surgery was 10.43 (±7.08) years. Subaortic membrane resection was the most commonly used technique (46.4%, n=13). It was associated with septal myomectomy in 8 patients (28.6%). Postoperative mortality rate was zero. Residual gradient ≥30 mmHg was reported in 8 patients (28.6%) after surgery. Recurrences were observed in 7 patients (25%) of whom 6 underwent reintervention. In multivariate analysis, only postoperative residual gradient was significantly associated with recurrence (p=0.030, OR=33.785, 95% CI: 1.398-816.754). Despite old age at diagnosis and surgery, favorable short-term outcomes were reported, but recurrences were frequent in the long term. This highlights the role of regular, perioperative and postoperative, clinical and echographic monitoring of these patients.

## Introduction

Les rétrécissements aortiques sous valvulaire congénitaux sont définis comme des obstacles fixes et permanents à la sortie du ventricule gauche (VG) situés en dessous des sigmoïdes aortiques. Ils couvrent un large éventail de lésions anatomiques, qui peuvent être subdivisées en deux catégories principales, les sténoses sous-aortiques discrètes et diffuses. Malgré qu´ils ne sont pas rares, représentant 8 à 20% de l´ensemble des obstacles de la voie d´éjection du ventricule gauche [1], ils sont souvent mal compris. Les rétrécissements aortiques sous valvulaire congénitaux sont considérés comme des lésions progressives, mais le degré de progression est variable d´un sujet à un autre [2]. En plus du risque de progression, les patients atteints d´un rétrécissement aortique sous valvulaire sont confrontés à un risque accru d´insuffisance aortique [3]. De ce fait, il y a peu de controverse en ce qui concerne la nécessité d'une intervention chirurgicale chez la plupart des patients atteints de sténose significative et/ou compliquée. Cependant, des controverses subsistent concernant le timing de la chirurgie et la technique chirurgicale. Bien que la résection chirurgicale précoce des membranes sous-aortiques avec ou sans myomectomie septale semble empêcher le développement de l´insuffisance aortique, la récidive reste une complication assez fréquente à long terme [4,5]. Un certain nombre de facteurs semblent influencer le risque de récidive. Notre travail a pour objectifs de décrire le profil épidémiologique et clinique des patients opérés pour rétrécissements aortiques sous valvulaire, rapporter l´expérience de notre centre en terme de prise en charge et de résultats post-opératoires et analyser les facteurs associés la récidive de l´obstacle sous aortique.

## Méthodes

**Type, cadre de l´étude**: il s´agit d´une étude rétrospective observationnelle portant sur tous les cas de rétrécissement aortique sous-valvulaire opérés et colligés dans le Service de Cardiologie de l'Hôpital Universitaire de Sfax entre le 01 janvier 2010 et le 31 décembre 2020.

**Population de l´étude**: nous avons inclus tous les patients opérés pour un obstacle sous-aortique membraneux et/ou musculaire, ou sous forme de tunnel ou secondaire à une anomalie d´insertion de la valve mitrale.

### Recueil des données

Le recueil de données cliniques et paracliniques a été effectué à partir des fiches et dossiers médicaux, des comptes rendus échocardiographiques et opératoires et par l´interrogatoire du patient ou son tuteur. Nous avons recueilli pour chaque patient les données démographiques à savoir l´âge au moment du diagnostic et le sexe, les données de l´examen clinique à savoir ses symptômes, les souffles auscultés et les autres anomalies de l´examen clinique, les anomalies observées à l´ECG et à la radiographie pulmonaire, les données échographiques pré-opératoires à savoir le type d´obstacle défini selon la classification de VOGT [6]: type l s´il s´agit d´une sténose en diaphragme membraneux, type ll s´il s´agit d´une sténose en bourrelet fibreux, type lll s´il s´agit d´une sténose fibro-musculaire diffuse et Type lV s´il s´agit d´un rétrécissement étendu en tunnel de toute la chambre de chasse du VG, la distance obstacle - valve aortique définie par la distance séparant la base de la membrane et l´insertion valvulaire aortique, le gradient maximal sous aortique, l´existence d´une éventuelle fuite aortique associée dont la sévérité a été évaluée en se basant sur les dernières recommandations de la societé européenne de cardiologie (ESC) [7], les malformations cardio-vasculaires associées et le retentissement cardiaque à savoir l´existence ou non d´une hypertrophie ventriculaire gauche (HVG) et/ou une altération de la fonction ventriculaire gauche systolique, les données opératoires à savoir l´âge au moment de la chirurgie, le délai entre le diagnostic et la chirurgie et la technique chirurgicale (une résection de la membrane sous aortique et/ou une myomectomie et les gestes associés) et les données échographiques post-opératoires à savoir l´existence ou non d´une fuite aortique, le gradient maximal sous aortique post-opératoire immédiat et à long terme. Nous avons considéré qu´il existe un gradient résiduel lorsque le gradient maximal post-opératoire immédiat est supérieur ou égal à 30 mmHg [8-10] et une récidive lorsque le gradient maximal augmente à long terme à une valeur supérieure ou égale à 50 mmHg [11]. En ce qui concerne le groupement, nous avons divisé l´âge au moment du diagnostic et au moment de la chirurgie, en 2 groupes ≤ 6 ans et > 6 ans. Nous avons classé la distance obstacle - valve aortique en 2 groupes; moins de 7 mm comme obstacle très proche de la valve aortique et supérieur ou égal à 7 mm comme distant de la valve, le gradient maximal sous aortique en 2 groupes: un gradient ≥ 50 mmHg en faveur une sténose significative nécessitant souvent un traitement chirurgical [12,13], ou < 50 mmHg traduisant souvent une sténose modérée et relève souvent d´une surveillance clinique et échographique. Nous avons classé le gradient résiduel en 2 groupes: modéré s´il est < 50 mmHg et significatif s´il est ≥ 50 mmHg [8-10].

### Analyse statistique.

Nous avons réalisé initialement une analyse descriptive en décrivant le profil épidémiologique, clinique thérapeutique et évolutif et de la population. Les données continues ont été présentées sous forme de moyenne et écart type alors que les variables catégorielles étaient présentées par des nombres et des pourcentages. Nous avons ensuite réalisé une analyse univariée suivie d´une analyse multivariée pour chercher les facteurs associés à la récidive de l´obstacle sous aortique. La comparaison des pourcentages sur séries indépendantes a été effectuée par le test du chi 2 de Pearson et la comparaison des données continues a été effectuée par le test de student. Nous avons utilisé pour l´analyse multivariée des modèles de régression logistique et nous avons inclus dans les modèles multivariés la classe d´âge, le sexe et toutes les variables ayant un indice de significativité p < 0,2 dans l´analyse univariée. Une valeur p inférieure à 0,05 était considérée comme significative. Les analyses statistiques ont été effectuées à l'aide du logiciel SPSS version 23.0 (IBM Corp., Armonk, NY, États-Unis).

**Considérations éthiques**: l'analyse des données s'est déroulée dans le respect des valeurs de l´éthique médicale, de l'anonymat et de la confidentialité. Un consentement oral préalable a été obtenu auprès de chaque patient participant à cette étude, ou son tuteur si le patient est mineur.

## Résultats

**Caractéristiques générales de la population**: pendant la période d´étude, nous avons relevé 157 cas d´obstacles de la voie d´éjection du VG dont 28 cas de rétrécissement aortique sous valvulaire, soit 17,83% des obstacles de la voie d´éjection du VG. Au moment du diagnostic, l´âge moyen était 6,82 (±4,84) ans et les symptômes étaient présents chez 19 patients (67,85%). A l´échocardiographie, un gradient maximal sous-aortique ≥ 50 mmHg était présent chez 23 patients (82,14 %). Les malformations cardiovasculaires associées à la sténose sous valvulaire étaient observées chez 16 patients (57,14%). Les différentes caractéristiques démographiques, cliniques et paracliniques de la population sont résumées dans le Tableau 1. Les différentes anomalies observées dans notre série sont illustrées dans le Tableau 2.

**Tableau 1:** caractéristiques générales de la population d´étude

Variables		N (%)
**Sexe**	**Masculin**	18(64,28)
	**Féminin**	10(35,71)
**Classe d´âge (au moment du diagnostic)**	**Age < 6 ans**	15(53,57)
	**Age ≥ 6 ans**	13(46,42)
**Symptômes**		19(67,85)
**Dyspnée d’effort**		15(53,57)
**Syncope d’effort**		2(7,14)
**Détresse respiratoire aiguë**		3(10,71)
**Asthénie d´effort**		4(14,28)
**Angor d´effort**		2(7,14)
**Souffle systolique**		28(100)
**Souffle diastolique**		15(53,57)
**HVG à l´ECG**		6(21,42)
**Cardiomégalie**		6(21,42)
**Surcharge hilaire**		3(10,71)
**Gradient max initial ≥ 50 mmhg**		23(82,14)
**HVG à l´échographie cardiaque**		12 (42,85)
**FEVG ≥ 60%**		27 (96,42)
**Type de l´obstacle**	**Type 1**	23 (82,14)
	**Type 2**	2(7,14)
	**Type 3**	3(10,71)
**Distance obstacle-valve aortique**	**≤7 mm**	8(28,57)
	**> 7 mm**	20(71,42)
**Insuffisance aortique**		21(75)
**Anomalies cardiovasculaires associées**		16 (57,14)

ECG: Électrocardiogramme ;FEVG :Fraction d´éjection du ventricule gauche; HVG: Hypertrophie ventriculaire gauche; N(%): Nombre et pourcentage des patients

**Tableau 2:** fréquence des malformations cardiovasculaires associées à la sténose aortique sous valvulaire dans la population d’étude

Anomalie	N(%)
**Sténose valvulaire congénitale (bicuspidie)**	5(17,85)
**Sténose sus-valvulaire**	1(3,58)
**IM significative**	1(3,58)
**Coarctation de l´aorte**	2(7,14)
**Dysplasie de la valve mitrale (excès du tissus)**	3(10,7)
**Canal artériel persistant**	2(7,14)
**CIV**	3(10,7)
**CIA**	1(3,58)
**Sténose pulmonaire**	1(3,58)

CIA: communication inter-atriale, CIV: communication interventriculaire, IM: insuffisance mitrale, N(%) : nombre et pourcentage des patients

**Prise en charge opératoire**: l´âge moyen au moment de la chirurgie était 10,43±7,08 ans. Le délai moyen de la chirurgie après le diagnostic de l´obstacle sous aortique était 3,44 ans [1 mois-14 ans]. Pour la grande majorité des patients (60,7%) l´indication a été établie dès la première consultation, alors que les autres patients ont été opérés secondairement après une aggravation du gradient maximal sous aortique (35,7%, n=10) et/ou apparition d´une hypertrophie ventriculaire gauche (HVG) à l´échographie cardiaque (10,71%, n=3) et/ou apparition de symptômes (7,14%, n=2). Le gradient maximal sous aortique préopératoire moyen était 78,07 ± 20,33 mmHg. La résection de la membrane sous-aortique était la technique la plus fréquemment utilisée (46,4%, n=13). Elle a été associée à une myomectomie septale chez 8 patients (28,6%) (Tableau 3).

**Tableau 3:** techniques chirurgicales utilisées dans la population d’étude

		N(%)
**Résection de membrane**	Sans autre geste associé	13 (46,4)
	Associée à une myomectomie	8(28,6)
	Associée à une plastie mitrale	1(3,6)
	Associée à une cure de coarctation	1(3,6)
	Associée à une fermeture de CIV	3(10,7)
	Associée à une fermeture de canal artériel	1(3,6)
**Myomectomie + fermeture de la CIV + élargissement de la voie pulmonaire**		1(3,6)

CIV : Communication interventriculaire, N (%) : nombre et pourcentage des patients

**Évolution post opératoire**: la mortalité opératoire était nulle. En post-opératoire immédiat, le gradient maximal sous aortique moyen était de 24,39 ±18,6 mmHg, soit une baisse de 68,75% par rapport au gradient maximal préopératoire. La présence d´un gradient résiduel a été notée chez 8 patients (28,6%). Une fuite aortique modérée (grade I ou II) a été observée chez 23 patients (82,1%). La durée du suivi post-opératoire était de 6,82 ± 4,15 ans (1 an-11 ans). La récidive de la sténose sous aortique a été observée chez 7 patients (25%) parmi lesquels 6 ont bénéficié d´une réintervention.

**Facteurs associés à la récidive de l´obstacle sous aortique à long terme**: dans l´analyse univariée (Tableau 4), les facteurs associés à la récidive était la présence d´un gradient maximal sous-aortique pré-opératoire ≥70 mmHg (nOR= 25, IC 95%: 2,35-275,73, p=0,001) et chez les patient ayant un gradient résiduel post-opératoire ≥30 mmHg (nOR= 57, IC 95% : 4,36-744,71, p< 0,001). Dans l´analyse multivariée (Tableau 4) après ajustement de ces deux paramètres échoghraphiques au sexe et l´âge au moment de la chirurgie, seul le gradient résiduel post opératoire était significativement associé à la récidive (aOR=33,785, IC 95% : 1,398- 816,754, p=0,030).

**Tableau 4:** analyse univariée et multivariée des facteurs associés à la récidive (gradient max ≥50 mmHg) à long terme chez les patients opérés

Variables			Analyse univariéé		Analyse multivariée	
		N (%)	nOR	p	aOR	p
**Sexe**	Masculin	4(22,2)		0,629		
	Féminin	3(30,0)			1,32(0,05-37,23)	0,870
**Classe d’âge (au moment de la chirurgie)**	≤ 6 ans	2(22,2)		0,815	1,78(0,06-49,04)	0,732
	> 6 ans	5 (26,3)				
**Délai de la chirurgie par rapport au diagnostic**	≤ 1 an	4(25)		1		
	> 1an	3(25)				
**Symptômes**	Oui	4(21,1)		0,483		
	Non	3 (33,3)				
**Gradient maximal pré-opératoire ≥ 70 mmHg**	Oui	6(60%)	25(2,35-275,73)	**0,001**	14,75(0,62-348,17)	0,095
	Non	1(5,6)				
**Type de l’obstacle**	Diaphragme	5 (21,7)		0,635		
	Fibro-musculaire	1(50,0)				
	Complexe	1(33,3)				
**Distance** **obstacle-valve aortique**	< 7 mm	1 (12,5)		0,334		
	≥ 7 mm	6 (30)				
**HVG**	Oui	4 (33,3)		0,378		
	Non	3(18,8)				
**Anomalies** **cardiovasculaires associées**	Oui	3(30,0)		0,649		
	Non	4 (22,2)				
**Type de la chirurgie \**	Myomectomie sans autre geste associ	4(30,8)		0,779		
	Myomectomie associée à une myomectomie	3 (37,5)				
	Myomectomie associée à une Plastie mitrale	0 (0)				
	Myomectomie associée à une Cure de coarctation	0 (0)				
	Myomectomie associée à une Fermeture de CIV	0 (0)				
	Myomectomie associée à une Fermeture de canal artériel	0 (0)				
	Myomectomie + fermeture de la CIV + élargissement de la voie pulmonaire	0 (0)				
	Intervention de Konno	0 (0)				
**Existence d’un gradient résiduel post opératoire**	Oui	6(75)	57	**<0,001**		
	Non	1(5)	(4,36-744,71)		33,78(1,39-816,75)	**0,030**

aOR : Odds Ratio ajusté, CIV : communication interventriculaire, N(%) : Nombre et pourcentage des patients, nOR :Odds ratio non ajusté, p : indice de significativité

## Discussion

Le rétrécissement aortique sous valvulaire, bien qu´il est peu fréquent, constitue la cardiopathie la plus fréquente des cardiopathies obstructives gauches après la sténose valvulaire [14]. A travers cette étude observationnelle, nous avons cherché à décrire les particularités cliniques et thérapeutiques des patients opérés pour un rétrécissement aortique sous valvulaire, évaluer les résultats opératoires à court et à long terme et identifier les facteurs associés la récidive post-opératoire de l´obstacle sous aortique. Au terme de cette étude, nous avons montré que malgré l´âge tardif des patients au moment du diagnostic et de la chirurgie, nos résultats étaient favorables à court terme. En revanche la récidive était fréquente à long terme, expliquée essentiellement par la persistance d´un gradient résiduel significatif chez certains patients. Dans notre série, le rétrécissement aortique sous valvulaire représente 17,83% des obstacles de la voie d´éjection du VG. Ce taux est proche des données de la littérature, ou il représente 8% à 30% des obstacles de la voie d´éjection du VG [8,9,15]. Il est considéré comme une maladie acquise se développant secondairement après la naissance [9] ce qui explique l´âge relativement tardif au moment du diagnostic et de la chirurgie tel est le cas dans notre série. Dans la littérature, l´âge au moment de la chirurgie varie selon les auteurs entre 3 et 18 ans [8-10,16].

La répartition de nos malades selon le sexe a montré une nette prédominance masculine avec un sexe ratio de 1,8. Cette prédominance masculine était observée dans plusieurs autres séries antérieures [8,9,16]. La plupart de nos patients étaient symptomatiques (67,5%). Nos résultats étaient différents comparativement aux résultats observés dans la littérature. Rayburn [17] et Darci [9] rapportent respectivement 39,1 et 30% de patients symptomatiques dans leurs séries. Conformément aux résultats de plusieurs études [10,16], le symptôme le plus fréquemment rapporté dans notre série était la dyspnée retrouvée chez 75% des patients symptomatiques. En plus du souffle systolique dû à l´obstacle sous aortique lui-même trouvé chez tous les patients, nous avons retrouvé un souffle diastolique d´insuffisance aortique dans 53,57% des cas. Dans la littérature, ce dernier a été rapporté chez 33% à 72% des patients [9,15,18]. L´insuffisance aortique, favorisée par les lésions du jet sur les sigmoïdes aortiques, est rapportée avec une fréquence allant de 54,1% [8] à 80,3% [10] selon les publications. Dans notre étude, elle a été observée dans 62,7% des cas. Le rétrécissement aortique sous valvulaire peut être isolé ou associé à d´autres malformations cardiaques. Ces dernières ont été observées dans 57,14% des cas de notre série. Il a été décrit comme une maladie acquise favorisée par des anomalies de la géomètrie de la voie d´éjection gauche créant des turbulences dans la région sous-aortique à l´origine des phénoménes de multiplication cellulaire [3]. Dans notre série, les anomalies les plus fréquemment obsevées étaient les communications interventriculaires (CIV) péri-membraneuses chez 3 patients, le canal artériel perméable chez 2 patients, la sténose valvulaire aortique sous forme de bicuspidie chez 5 patients, la sténose sus-valvulaire chez un patient et la coarctation chez 2 patients. Nos résultats étaient proches des données de la littérature dans lesquelles la CIV, la coarctation de l´aorte, la persistance du canal artériel et la bicuspidie aortique étaient les anomalies les plus observes [3]. Le mécanisme physiopathologique commun pour ces différentes anomalies était l´hyper-débit et/ou l´hyperpression au niveau de la région sous-aortique entrainant des anomalies de différenciation cellulaire et favorisant la genèse d´un obstacle sous-aortique [19,20].

Dans notre série, pour la grande majorité (60,7%) l´indication opératoire était établie dès la première consultation. La nécessité d´un gradient sous-aortique significatif avant la chirurgie est toujours recommandée par la plupart des centres mais une chirurgie plus rapide est souvent indiquée pour les nourrissons et les enfants quelque soit le gradient. La stratégie s´est appuyée dans la plupart des series [9,10], conformément aux recommandations européennes et américaines [12,21] sur 3 éléments essentiels: 1) un gradient maximal sous-aortique supérieur à 50 mmHg; 2) une fuite aortique progressive; 3) une lésion associée nécessitant une cure sous circulation extracorporelle. En plus de ces critères, plusieurs autres éléments doivent être pris en considération avant de poser l´indication chirurgicale, à savoir l´âge du patient lors du diagnostic et le degré du retentissement de la cardiopathie sur le plan fonctionnel et hémodynamique. La chirurgie des sténoses sous aortiques peut être très simple, comme elle peut être très complexe [16]. La résection chirurgicale de la membrane reste la technique de choix. Actuellement, ce geste s´accompagne de plus en plus souvent d´une myomectomie du septum afin d´élargir la voie sous-aortique en avant [15,22]. L´association d´une myomectomie septale a permis pour plusieurs auteurs de réduire considérablement aussi bien le gradient sous-aortique postopératoire immédiat que le taux de récidive [23]. La résection de membrane simple était la technique la plus fréquemment utilisée dans notre série, pratiquée chez prés de la moitié des patients opérés. Elle a été associée à une myomectomie septale chez 8 patients (28,6% des patients) et à une cure chirurgicale des anomalies cardiovasculaires associées chez 7 patients (25% des patients).

Dans notre série, le gradient post opératoire a baissé de 68,75%, et 8 patients (28,6%) présentaient en post-opératoire un gradient maximal sous-aortique >30 mm Hg. Ces résultats immédiats étaient proches aux résultats observés dans la majorité des études antérieures dans lesquelles la baisse du gradient maximal varie de 63% à 82,7% [8,9,15]. La persistance d´un gradient résiduel en post-opératoire peut être en rapport avec une résection incomplète ou une anomalie obstructive complexe de la voie de sortie gauche. Pour le devenir de l´insuffisance aortique, les conséquences de la levée chirurgicale de l´obstacle sous aortique sont très diversement rapportées. Certains auteurs décrivent une stabilisation voir une amélioration, d´autres au contraire une aggravation après chirurgie [24], dans notre série, une fuite aortique modérée (grade I ou II) a été observée chez 23 patients (82,1%). La récidive du rétrécissement aortique sous valvulaire a été observée chez 25% de nos patients. Ce taux était proche des taux observés dans les différentes séries publiées dans lesquelles il varie entre 14 à 27% [5,25,26]. Dans notre série, la récidive était corrélée à l´importance du gradient sous-aortique pré-opératoire et l´existence d´un gradient résiduel post-opératoire. Dans la littérature, les facteurs associés à la récidive étaient la forme anatomique complexe, la résection musculaire initiale insuffisante, l´existence d´un gradient résiduel sous-aortique post opératoire ≥ 30 mmHg et la présence d´ anomalies valvulaires aortiques associées [16,25,27] . La principale faiblesse de notre étude est qu´il s´agit d´une étude rétrospective avec un nombre limité de patients (n=28). D´autre part, la lésion principalement traitée est la membrane sous-aortique (82,14%, n=23), alors que les autres types d´obstacles sous-valvulaires sont très peu représentés dans notre série. L´ensemble de ces éléments limitent la généralisation de nos résultats.

## Conclusion

Le rétrécissement aortique sous valvulaire est une cardiopathie peu fréquente. Son diagnostic se fait souvent à l´occasion d´une auscultation cardiaque pathologique d´un souffle systolique chez des enfants souvents asymptomatiques. Il repose sur l´échographie cardiaque qui objective un diaphragme membraneux chez la plupart des patients, souvent associé à une insuffisance aortique modérée et à d´autres malformations cardiovasculaires. Son traitement est toujours chirurgical, conservant la valve aortique, consiste en une résection de l´obstacle sous-aortique. Le moment de la chirurgie est conditionné par l´âge du patient, le retentissement clinique et hémodynamique de l´obstacle, le type anatomique d´obstacle et les lésions associées. Malgré l´âge tardif des patients au moment du diagnostic et de la correction, nos résultats étaient favorables à court terme sur le plan fonctionnel et hémodynamique. Il n´y avait aucune mortalité opératoire dans notre série. En revanche, le traitement chirurgical est rarement définitif vu que la récidive est fréquente, observée chez 25% de nos patients, expliquée essentiellement par la persistance d´un gradient résiduel significatif en post opératoire immédiat chez certains patients. Nos résultats soulignent l´intérêt d´assurer un diagnostic précoce et suivi rigoureux, clinique et échographique pré et postopératoire de ces patients.

### Etat des connaissances sur le sujet

- *Le rétrécissement aortique sous valvulaire est une cardiopathie très polymorphe et évolutive;*
- *L´évolution spontanée du rétrécissement aortique sous valvulaire se fait vers l´aggravation du gradient et vers l´apparition de complications en particulier une insuffisance aortique d´aggravation progressive imposant toujours un traitement chirugical. Plusieurs controverses existent concernant le timing de la chirurgie et la technique chirurgicale;*
- *La récidive reste une complication assez fréquente à long terme due à un certain nombre de facteurs encore mal élucidés*.

### Contribution de notre étude à la connaissance

- *Nous avons démontré que la résection chirurgicale précoce des membranes sous-aortiques, idéalement associée à une myomectomie septale empêche le développement de l´insuffisance aortique et réduit considérablement le gradient sous-aortique post-opératoire immédiat ainsi que le risque de rêcidive à long terme;*
- *Vu le potentiel évolutif du rétrécissement aortique sous valvulaire, un suivi régulier, clinique et échographique attentif s´impose avant la chirurgie à fin de poser l´indication opératoire au moment opportun, mais aussi après la chirurgie*.
